# The Effects of Flavonol and Flavone Glucuronides from *Potentilla chinensis* Leaves on TNF-α-Exposed Normal Human Dermal Fibroblasts

**DOI:** 10.3390/antiox12101803

**Published:** 2023-09-27

**Authors:** Yea Jung Choi, So Young Lee, So-Ri Son, Jun Yeon Park, Dae Sik Jang, Sullim Lee

**Affiliations:** 1 College of Korean Medicine, Gachon University, Seongnam 13120, Republic of Korea; domdada22@gachon.ac.kr; 2 Department of Biomedical and Pharmaceutical Sciences, Graduate School, Kyung Hee University, Seoul 02447, Republic of Korea; thdudrhswl@khu.ac.kr (S.Y.L.); allosori@khu.ac.kr (S.-R.S.); 3 Department of Food Science and Biotechnology, Kyonggi University, Suwon 16227, Republic of Korea; 4 Department of Life Science, College of Bio-Nano Technology, Gachon University, Seongnam 13120, Republic of Korea

**Keywords:** flavonol glucuronide, flavone glucuronide, potentilloside A, ROS, MMP-1, COLIA1

## Abstract

Skin aging is a complex biological process influenced by a variety of factors, including UV radiation. UV radiation accelerates collagen degradation via the production of reactive oxygen species (ROS) and cytokines, including TNF-α. In a prior investigation, the inhibitory properties of flavonol and flavone glucuronides derived from *Potentilla chinensis* on TNF-α-induced ROS and MMP-1 production were explored. Consequently, we verified the skin-protective effects of these flavonol and flavone glucuronides, including potentilloside A, from *P*. *chinensis,* and conducted a structure–activity relationship analysis as part of our ongoing research. We investigated the protective effects of the extract and its 11 isolates on TNF-α-stimulated normal human dermal fibroblasts (NHDFs). Ten flavonol and flavone glucuronides significantly inhibited ROS generation (except for **7**) and suppressed MMP-1 secretion, except for **2**. In contrast, six isolates (**1**, **5**, **6**, **11**, **9**, **10**, and **11**) showed a significant reverse effect on COLIA1 secretion. Comparing the three experimental results of each isolate, potentilloside A (**1**) showed the most potent skin cell-protective effect among the isolates. Evaluation of the signaling pathway of potentilloside A in TNF-α-stimulated NHDF revealed that potentilloside A inhibits the phosphorylation of ERK, JNK, and c-Jun. Taken together, these results suggest that compounds isolated from *P. chinensis*, especially potentilloside A, can be used to inhibit skin damage, including aging.

## 1. Introduction

Skin aging is a complex biological process that is influenced by several factors. Aging is divided into two types: intrinsic and extrinsic aging. Intrinsic aging is programmed aging caused by interior factors in the human body. Extrinsic aging is caused by environmental factors, such as smoking, pollution, and UV radiation [1]. UV radiation, a common extrinsic factor, accelerates the breakdown of collagen in the skin, a critical component of the extracellular matrix (ECM). Collagen degradation contributes to visible signs of aging, including wrinkles and sagging [2].

UV radiation exposure triggers the release of tumor necrosis factor-α (TNF-α), a cytokine involved in the inflammatory response of the skin. TNF-α exacerbates skin inflammation and plays a role in the aging process. UV radiation induces the production of reactive oxygen species (ROS) in skin cells. ROS are highly reactive molecules that contribute to oxidative stress and are significant factors in skin aging [3,4]. A positive feedback loop exists between TNF-α and ROS production. TNF-α stimulates ROS generation, which can activate signaling pathways that promote TNF-α production and intensify the inflammatory response [5,6].

Both ROS and TNF-α activate matrix metalloproteinases (MMPs). MMPs, particularly MMP-1, are enzymes responsible for collagen degradation within the ECM. Collagen breakdown leads to visible skin-aging effects. Consequently, MMP-1 and proinflammatory cytokines also play a role in age-related skin conditions, such as psoriasis, acne vulgaris, and atopic dermatitis [7,8,9]. ROS upregulate various signaling pathways, such as MAPKs, AP-1, and NF-κB, which are associated with MMP-1 production and proinflammatory cytokines, contributing to skin aging [10].

Hence, the inhibition of oxidative stress and TNF-α activity holds significant potential as a promising approach for the creation of novel therapeutic agents aimed at enhancing skin aging or managing inflammatory skin conditions.

The genus Potentilla contains approximately 500 known species, some of which are associated with the Latin word “potens”, which means “strong”. *Potentilla chinensis* is widespread throughout East Asia, particularly in China and Korea [11]. Previous studies have highlighted the antioxidant and anti-inflammatory properties of this plant [12].

In a previous study, we isolated and identified 11 compounds, including the new compound potentilloside A, from *P. chinensis* leaves and discovered their potential biological activity. All isolates were tested for the inhibition of TNF-α-induced ROS at each concentration, with no effect on cell proliferation or inhibition, and the isolates showed a significant inhibitory effect. Among the isolates, four flavonol-bis-glucuronides, which are rare in nature and whose pharmacological activity is not well known, were tested for their MMP-1 inhibitory effect, and potentilloside A and quercetin-bis-3,7-*O*-*β*-d-glucuronide showed significant inhibition [13]. Thus, the possibility of new activities of the two structurally rare compounds was suggested.

In this study, as part of ongoing research, we aimed to determine the skin-protective effects of flavonol and flavone glucuronides from *P. chinensis* and to analyze the structure–activity relationship. In addition, we will identify the compounds with the strongest effects and propose them as representative active ingredients through mechanistic studies.

## 2. Materials and Methods

### 2.1. Cell Culture

Normal human dermal fibroblasts (NHDFs) were acquired from PromoCell GmbH (Heidelberg, Germany). NHDFs were cultured in DMEM (Corning, Manassas, VA, USA) containing FBS (10%, *v*/*v*) (Atlas, Fort Collins, CO, USA) and 100 U/mL of antibiotics (penicillin–streptomycin, Gibco, Grand Island, NY, USA) at 37 °C and incubated in a 5% CO_2_ atmosphere; the cells within passage numbers 6–10 were used for subsequent experiments.

### 2.2. Cell Viability

NHDFs were plated in 96-well flat-bottomed microplates (5 × 10^3^ cells/well). The indicated concentrations of the extract and all the isolates were treated for 24 h. A cell viability assay was conducted using the Ez-Cytox solution. Briefly, 10% Ez-Cytox solution was added into each well and reacted at 37 °C for 1 h. Viability was measured using a microplate reader at 450 nm.

### 2.3. Intracellular ROS Generation Assay

NHDFs were plated in 48-well flat-bottomed microplates (1 × 10^4^ cells/well). NHDFs were pre-treated with the indicated concentrations of isolates for 1 h. Next, the cells were treated with 20 ng/mL TNF-α and 10 μM DCF-DA (2,7-dichlorofluorescein diacetate) for 15 min in 48-well plates. Next, the medium was removed, and the cells were washed with DPBS (Welgene, Gyeongsan, Republic of Korea). Cells were stained with DCFDA and visualized under a fluorescence microscope (Olympus, Tokyo, Japan). To quantify intracellular ROS generation levels, cells were seeded on a 96-well black plate (5 × 10^3^ cells/well) and treated with the concentrations mentioned above. Relative fluorescence intensities were measured using a microplate reader (SPARK 10M; Tecan, Männedorf, Switzerland) at excitation and emission wavelengths of 485/535 nm. The levels of intracellular ROS were expressed as fold changes.

### 2.4. Enzyme-Linked Immunosorbent Assay (ELISA)

NHDFs were cultured in 48-well plates (2 × 10^4^ cells/well) for 24 h in DMEM. After 24 h, the cells were starved overnight in serum-free DMEM. Eleven isolates and extract were added at the indicated concentrations (1, 3, 10, 30, and 100 μM or μg/mL) and incubated for 1 h. TNF-α was then added for 24 h to measure MMP-1 and COLIA1 secretion. Commercial ELISA kits (R&D Systems, Minneapolis, MN, USA) were used to quantify MMP-1 and COLIA1 concentrations, according to the manufacturer’s protocol. The absorbance at 450 nm was measured using a microplate reader.

### 2.5. Western Blotting

NHDFs were incubated in 6-well microplates (1.53 × 10^5^ cells/well) in complete DMEM and incubated for 24 h (37 °C, 5% CO_2_). After incubation, cells were treated with **1** at concentrations of 10, 30, and 100 µM for 1 h. After 1 h, the cells were treated with 20 ng/mL of TNF- for 24 h. After 24 h, the cells were lysed by scraping with RIPA buffer to collect the proteins. The mixture was sonicated and centrifuged at 14,000 rpm for 10 min at 4 °C. The protein concentration was determined using BCA protein method. Proteins were separated using a 4–20% premade protein gel (SDS-PAGE) and transferred to PVDF membranes (Bio-Rad, Hercules, CA, USA). Membranes were blocked with 5% nonfat milk in Tris-buffered Tween20 for 1 h and incubated with primary antibodies (ERK 1/1000, JNK 1/1000, p38 1/1000, p65 1/1000, c-jun 1/1000, c-fos, and GAPDH cell signaling) overnight at 4 °C. Membranes were re-incubated with HRP-conjugated secondary antibody (Anti-rabbit 1/5000, Cell signaling, Danvers, MA, USA) for 1 h. Membranes were developed using an enhanced chemiluminescence (ECL) plus reagent (Bio-Rad, Hercules, CA, USA).

### 2.6. Statistical Analysis

Data are expressed as means ± standard deviation (SD). Statistical significance was determined via one-way analysis of variance (ANOVA) using GraphPad Prism version 9. Tukey’s multiple comparison test was used to assess the differences between groups, with statistical significance set at *p* < 0.05.

### 2.7. Sample Preparation

**Figure 1:**
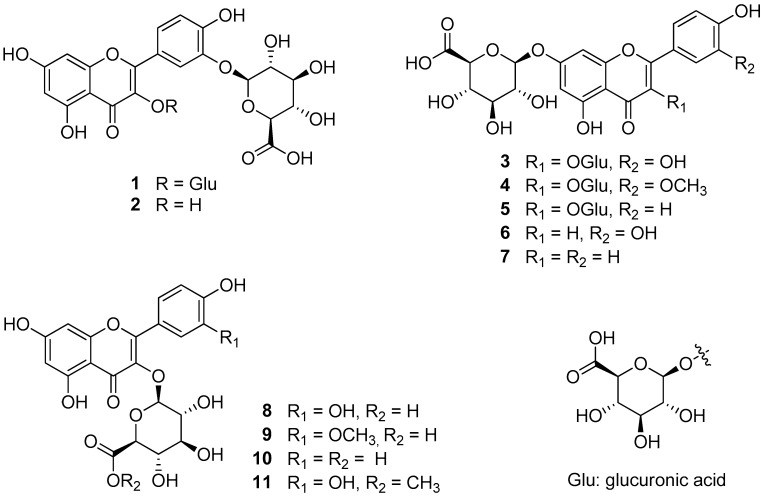
Chemical structures of isolates **1**–**11** from the leaves of *P. chinensis*.

The leaves of *P. chinensis* were collected in August at Evada Botanical Garden (GPS coordinates: X: 126.926768 and Y: 37.2482236) and dried for extraction. Four flavonol-di-glucuronides (**1** and **3***–***5**), five flavonol-mono-glucuronides (**2** and **8***–***11**), and two flavone-mono-glucuronides (**6** and **7**) were isolated from the 30% ethanol extract of *P*. *chinensis* leaves (Figure 1) [13].

The chemical structures were determined by comparing their spectral data with those reported in the literature as quercetin-di-3,3′-*O*-*β*-d-glucuronide (potentilloside A, **1**), quercetin-3′-*O*-*β*-d-glucuronide (**2**), quercetin-di-3,7-*O*-*β*-d-glucuronide (**3**), isorhamnetin-di-3,7-*O*-*β*-d-glucuronide (**4**), kaempferol-di-3,7-*O*-*β*-d-glucuronide (**5**), luteolin-7-*O*-*β*-d-glucuronide (**6**), apigenin-7-*O*-*β*-d-glucuronide (**7**), quercetin-3-*O*-*β*-d-glucuronide (**8**), isorhamnetin-3-*O*-*β*-d-glucuronide (**9**), kaempferol-3-*O*-*β*-d-glucuronide (**10**), and quercetin-3-*O*-*β*-d-glucuronide-6″-methyl ester (**11**).

## 3. Results

### 3.1. Effects of the Extract and Isolates on NHDF Viability

**Figure 2:**
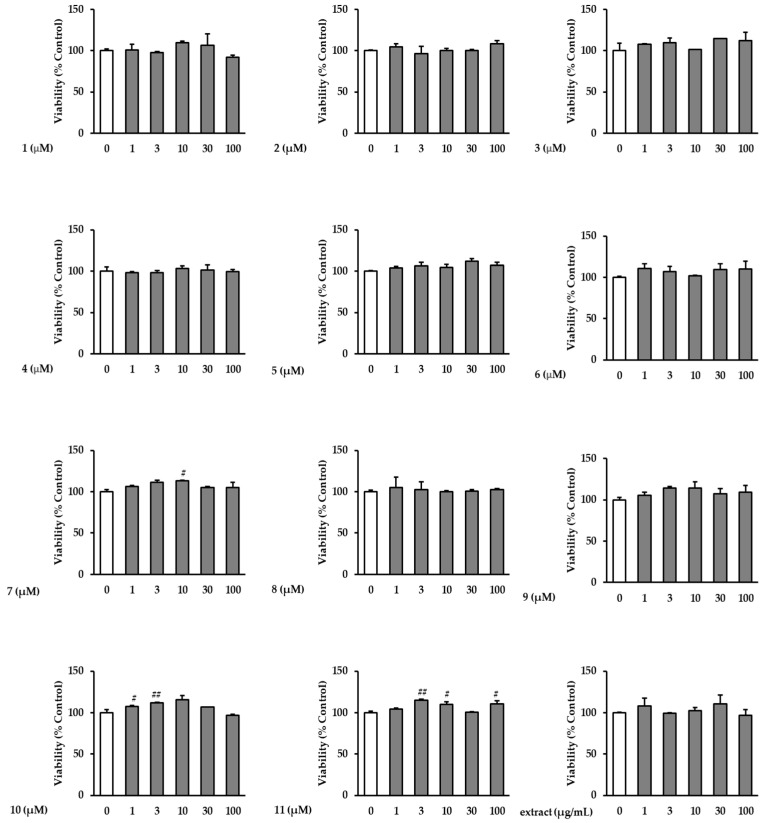
Effects of the extract and isolates on the viability of NHDFs. NHDFs were treated with the indicated concentrations of the extract and isolates for 24 h, and cell viability was evaluated using EZ-Cytox reagent kits. Data are presented as triplicate experiments. ^##^ *p* < 0.01 and ^#^ *p* < 0.05 versus the non-treated group.

To evaluate the protective effects of the extract and isolates on TNF-α-induced NHDFs in the non-cytotoxic range, a viability assay was conducted. In the concentration range of 1–100 μM, the extract and isolates did not show remarkable cytotoxicity toward NHDFs (Figure 2).

### 3.2. Effects of the Extract and Isolates on Intracellular ROS Generation in TNF-α-Stimulated NHDFs

**Figure 3:**
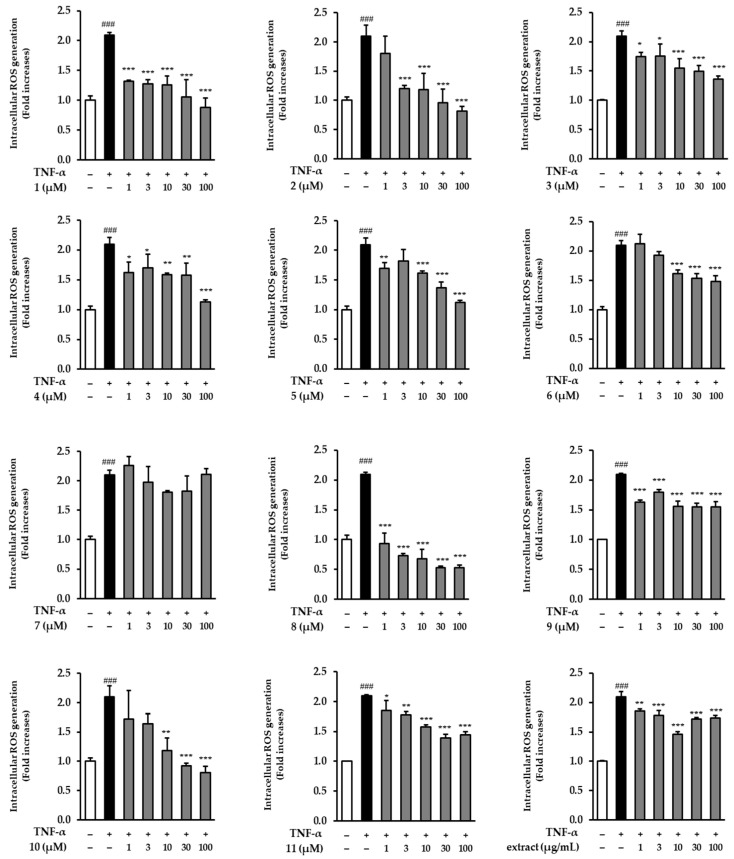
Inhibitory effect of the extract and isolates on intracellular ROS generation in TNF-α-induced NHDFs. NHDFs were seeded in a black 96-well plate at a density of 1 × 10^4^ cells per well and allowed to incubate for 24 h. The medium was replaced with serum-free DMEM under serum-free conditions to arrest the cell cycle. Subsequently, the indicated concentrations of *P. chinensis* extract and isolates were treated for 1 h, followed by continuous co-treatment with 20 ng/mL TNF-α and 10 µM dichlorofluorescin diacetate (DCFDA) for 15 min. Data are presented as mean ± SD (*n* = 3). ^###^ *p* < 0.001 versus the non-treated group; *** *p* < 0.001, ** *p* < 0.01 and * *p* < 0.05 versus the TNF-α-treated group.

Next, we determined the inhibitory effect of the extract and isolates on ROS generation in TNF-α-induced NHDFs at the same concentrations (1, 3, 10, 30, and 100 μM) in Figure 3. All isolates inhibited ROS generation in TNF-α-treated NHDFs; particularly, compound **1** significantly inhibited the ROS generation at all tested concentrations (1–100 μM) to 1.32 ± 0.02-fold (*p* < 0.001), 1.27 ± 0.08-fold (*p* < 0.001), 1.25 ± 0.15-fold (*p* < 0.001), 1.06 ± 0.29-fold (*p* < 0.001), and 0.88 ± 0.16-fold (*p* < 0.001) compared to the TNF-α-treated group (2.1 ± 0.04-fold, *p* < 0.001). Additionally, compound **8** significantly decreased ROS generation at all treatment dosages compared with that of the TNF-α-treated group. In addition, compound **2** diminished ROS generation to 0.96 ± 0.23-fold (*p* < 0.001) and 0.82 ± 0.08-fold (*p* < 0.001) at 30 and 100 μM, respectively. Compound **10** decreased ROS generation to 0.93 ± 0.04-fold (*p* < 0.001) and 0.80 ± 0.11-fold (*p* < 0.001) at 30 and 100 μM, respectively.

### 3.3. Effects of the Extract and Isolates on MMP-1 Secretion in TNF-α-Stimulated NHDFs

**Figure 4:**
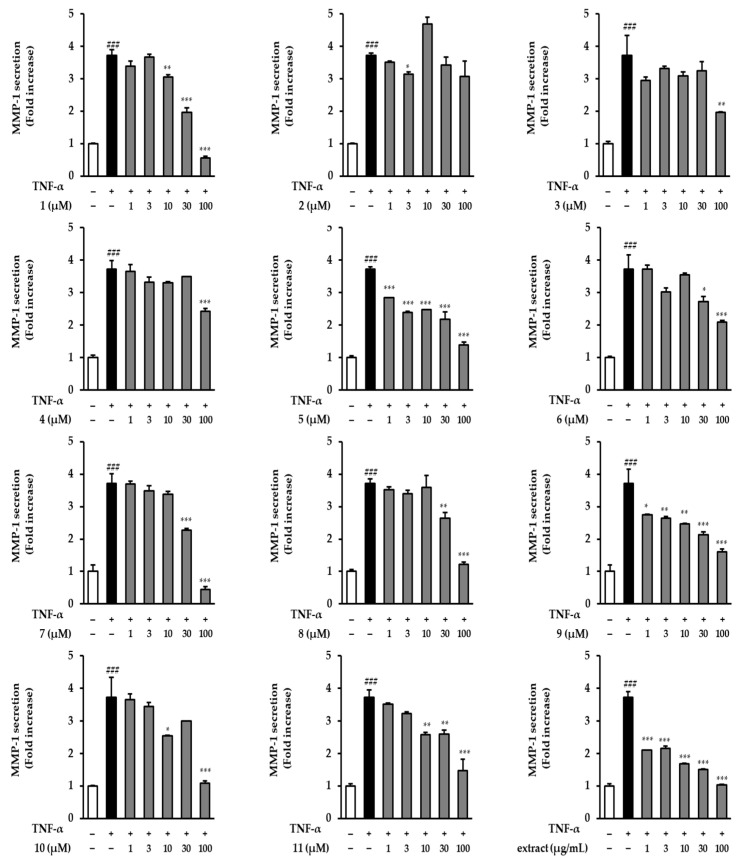
Effects of the extract and isolates on MMP-1 protein expression in TNF-α-stimulated NHDFs. NHDFs were seeded in a 48-well plate at a density of 2 × 10^4^ cells per well and allowed to incubate for 24 h. The medium was replaced with serum-free DMEM for serum-starved conditions to arrest the cell cycle. Subsequently, the extract and isolates were treated with the specified concentrations for 1 h, followed by continuous treatment with 20 ng/mL TNF-α for 24. h. MMP-1 secretion was quantified using an ELISA kit. Data are presented as mean ± SD (*n* = 2). ^###^ *p* < 0.001 versus the untreated group; *** *p* < 0.001, ** *p* < 0.01, and * *p* < 0.05, versus the TNF-α-treated group.

ELISA was conducted to determine the inhibitory effects of the extract and isolates on MMP-1 secretion in TNF-α-treated NHDFs. As shown in Figure 4, the extract and all the isolates inhibited MMP-1 secretion in TNF-α-induced NHDFs. Ten flavonol and flavone glucuronides, except for compound **2**, significantly inhibited the MMP-1 secretion at 100 μM. The extract caused significant concentration-dependent suppression at all concentrations. Treatment with 30 and 100 μM of compound **1** dramatically (potentilloside A) decreased MMP-1 secretion by 1.96 ± 0.14-fold (*p* < 0.001) and 0.56 ± 0.05-fold (*p* < 0.001), respectively, compared to the TNF-α group (3.72 ± 0.17-fold, *p* < 0.001). Additionally, compounds **5** and **9** significantly decreased MMP-1 secretion at all tested treatment concentrations. Furthermore, compounds **7** and **10** significantly inhibited MMP-1 secretion at 100 μM to 0.45 ± 0.08-fold (*p* < 0.001) and 1.09 ± 0.06-fold (*p* < 0.001), respectively.

### 3.4. Effects of the Extract and Isolates on COLIA1 Secretion in TNF-α-Stimulated NHDFs

**Figure 5:**
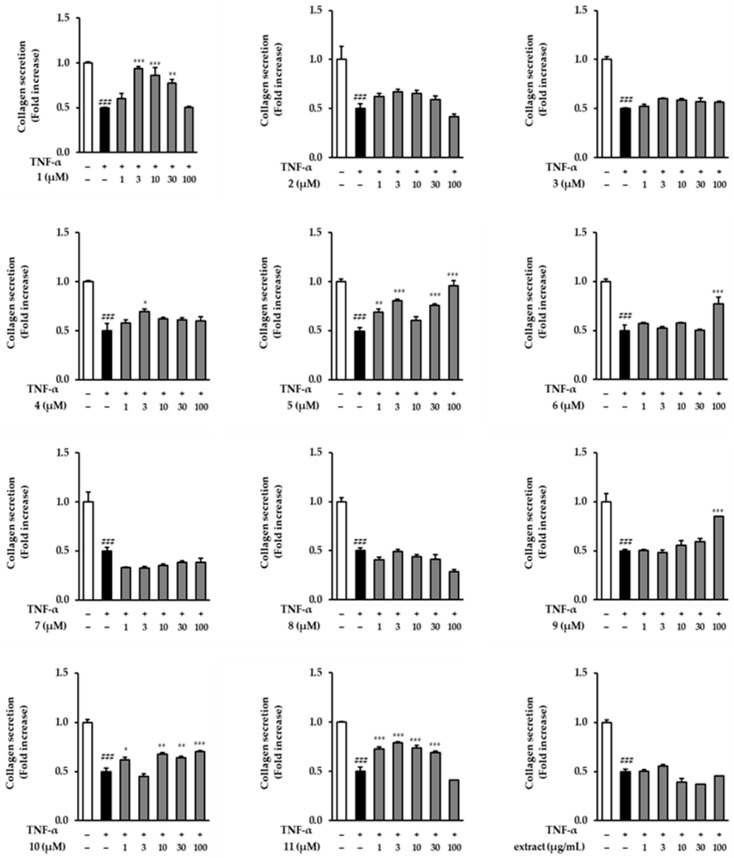
Effect of the extract and isolates on COLIA1 (pro-collagen 1) protein expression in TNF-α-stimulated NHDFs. NHDFs were seeded in a 48-well plate at a density of 2 × 10^4^ cells per well and allowed to incubate for 24 h. The medium was replaced with serum-free DMEM for serum-starved conditions to arrest the cell cycle. Subsequently, the extract and isolates were treated with the specified concentrations for 1 h, followed by continuous treatment with 20 ng/mL TNF-α for 24. h. COLIA1 secretion was quantified using an ELISA kit. Data are presented as mean ± SD (*n* = 2). ^###^ *p* < 0.001 versus the untreated group; *** *p* < 0.001, ** *p* < 0.01, and * *p* < 0.05, versus the TNF-α-treated group.

Next, we evaluated COLIA1 secretion in the TNF-α-treated condition. As shown in Figure 5, several compounds (**1**, **4**, **5**, **6**, **9**, **10**, and **11**) significantly increased COLIA1 secretion at different concentrations. However, the extract did not show an increase compared to that in the TNF-α-treated group. Compound **1** at 3, 10, and 30 µM potently increased COLIA1 secretion to 0.94 ± 0.02-fold (*p* < 0.001), 0.86 ± 0.09-fold (*p* < 0.001), and 0.77 ± 0.04-fold (*p* < 0.01), respectively, compared to the TNF-α-treated group (0.50 ± 0.00-fold, *p* < 0.001). In particular, 100 µM of compound **5** almost recovered the COLIA1 secretion by 0.96 ± 0.05-fold (*p* < 0.001).

### 3.5. Spider Chart for Efficiency Comparison of the Extract and Isolates in TNF-α-Stimulated NHDFs

A spider chart was used to evaluate the following three factors: inhibition of MMP-1 secretion, inhibition of ROS generation, and stimulation of COLIA1 expression. The rates of inhibition or increase at each concentration were calculated by converting the difference between the TNF-α-treated group and the untreated group to 100%. These were then assigned as follows: 0 (x < 20%), 1 (20 ≤ x < 40%), 2 (40 ≤ x < 60%), 3 (60 ≤ x < 80%), and 4 (80 ≤ x < 100%). The scores for the three experiments were then summed up. The total scores were divided into three ranges: 0 (y ≤ 4), 1 (5 ≤ y ≤ 9), 2 (10 ≤ y ≤ 14), and 3 (15 ≤ y).

**Figure 6:**
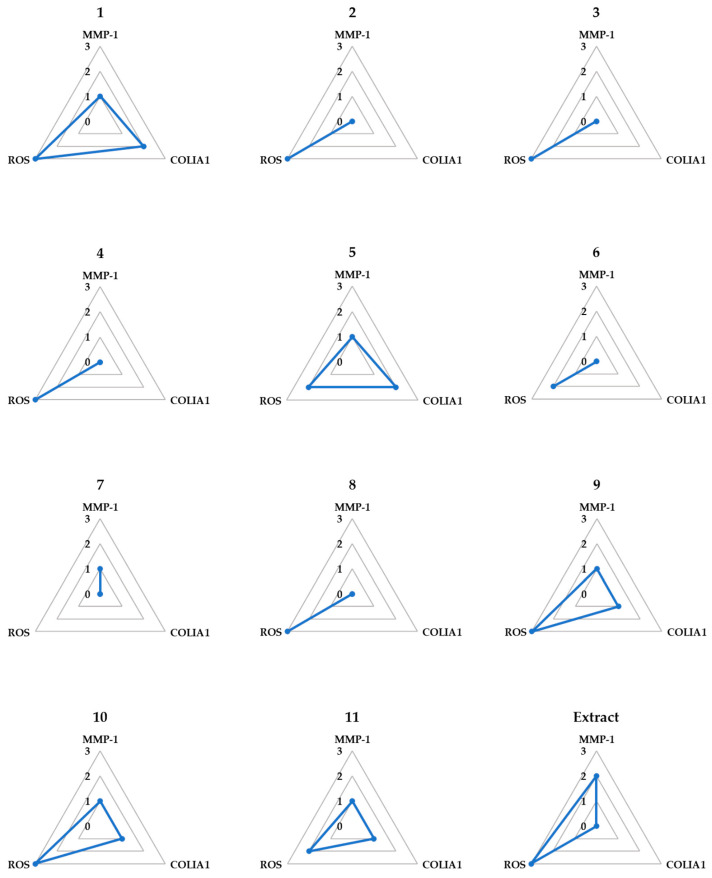
Spider chart for efficiency comparison of the isolates and extract on three markers associated with TNF-α-induced NHDF damage. The three markers included inhibition of MMP-1 secretion, inhibition of ROS generation, and stimulation of COLIA1 secretion. The scores assigned to the 12 tested samples ranged from three, representing the strongest effect, to one, indicating the lowest effect, for each factor. Additionally, a score of zero was assigned when no effect was observed for a particular factor.

As shown in Figure 6, compound **1** had the highest overall score, followed by compounds **5**, **9**, and **10**, respectively. Notably, compounds **1**, **5**, **7**, **9**, **10**, and **11** exhibited MMP-1 suppression. Among the flavonols with a glucuronide moiety at position 3, compounds **1** and **5** obtained the highest scores for stimulating COLIA1, followed by **9**, **10**, and **11**. All compounds, except compound **7**, demonstrated substantial ROS-inhibiting activity. Consequently, according to the spider chart evaluation, potentilloside A (**1**) was identified as the most effective compound.

### 3.6. Effects of Potentilloside A (***1***) on Intracellular ROS Generation in TNF-α-Stimulated NHDFs

**Figure 7:**
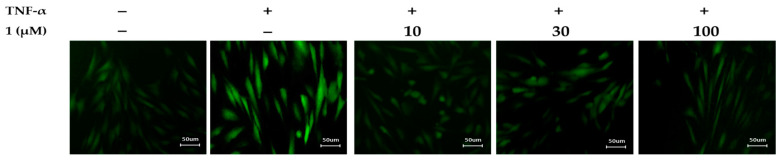
Effects of potentilloside A (**1**) on intracellular ROS generation in TNF-α-stimulated NHDFs. NHDFs were seeded in 48-well plate for 24 h. The medium was replaced with serum-free DMEM for serum-starved conditions to arrest the cell cycle. The cells were then treated with the indicated concentrations of **1** for 1 h. Subsequently, 20 ng/mL TNF-α and 10 µM DCFDA were added to each well for 15 min. Cells stained with DCF-DA were observed under a fluorescence microscope (×50).

To clarify the inhibitory effect of potentilloside A (**1**) on intracellular ROS generation in TNF-α-induced NHDFs, the cells were visualized using a DCFDA staining assay. As shown in Figure 7, potentilloside A (**1**) conspicuously inhibited fluorescence, indicating ROS generation, compared to that of the TNF-α-treated group.

### 3.7. Effect of Potentilloside A (***1***) on MAPK Phosphorylation in TNF-α-Stimulated NHDFs

**Figure 8:**
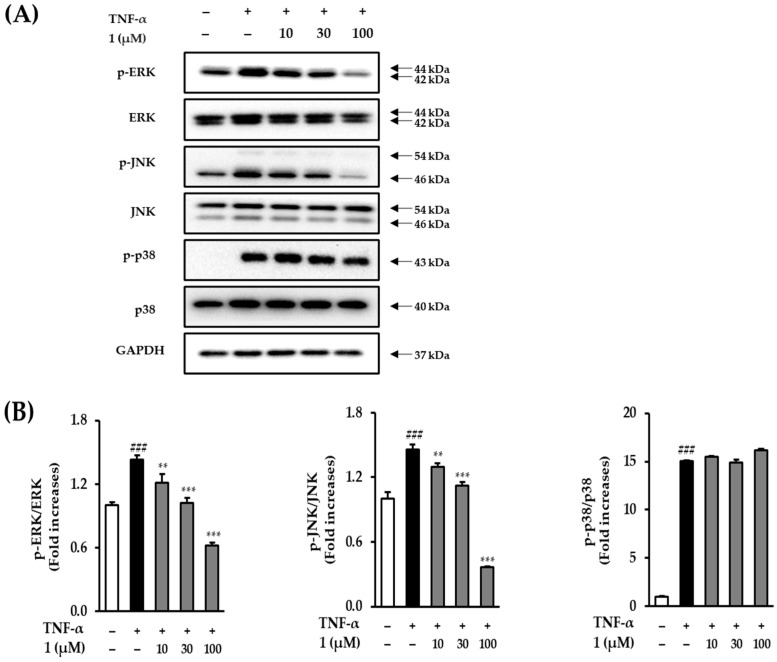
Effects of potentilloside A (**1**) on TNF-α-induced MAPK phosphorylation in NHDFs. (**A**) NHDFs were treated with 10, 30, and 100 μM potentilloside A (**1**) for 1 h and then with 20 ng/mL TNF-α for 15 min. Immunoreactive bands were analyzed by immunoblotting for p-JNK, JNK, p-ERK, ERK, p-p38, p38, and GAPDH. (**B**) The levels of p-p38, p-JNK, and p-ERK are expressed as the ratio of phosphorylated proteins to the corresponding total proteins. Data are presented as mean ± SD (*n* = 3). ^###^ *p* < 0.001 vs. control. ** *p* < 0.01 and *** *p* < 0.001 vs. TNF-α-exposed group.

Western blotting was performed to ascertain whether potentilloside A (**1**) plays a critical role in TNF-α-induced MAPK phosphorylation in NHDFs to analyze the MAPK biomarkers. As shown in Figure 8, an increase in ERK phosphorylation was observed in NHDFs exposed to TNF-α (1.43 ± 0.04-fold, *p* < 0.001). In contrast, treatments of 10, 30, and 100 μM potentilloside A (**1**) significantly decreased the phosphorylation of ERK to 1.21 ± 0.08-fold (*p* < 0.01), 1.02 ± 0.05-fold (*p* < 0.001), and 0.62 ± 0.03-fold (*p* < 0.001), respectively. TNF-α treatment increased JNK phosphorylation by 1.46 ± 0.04-fold (*p* < 0.001), and 30 and 100 μM potentilloside A (**1**) markedly decreased it by 1.12 ± 0.03-fold (*p* < 0.001) and 0.37 ± 0.01-fold (*p* < 0.001), respectively. However, potentilloside A (**1**) failed to inhibit p38 phosphorylation.

### 3.8. Effect of Potentilloside A (***1***) on NF-κB and c-Jun Phosphorylation in TNF-α-Stimulated NHDFs

**Figure 9:**
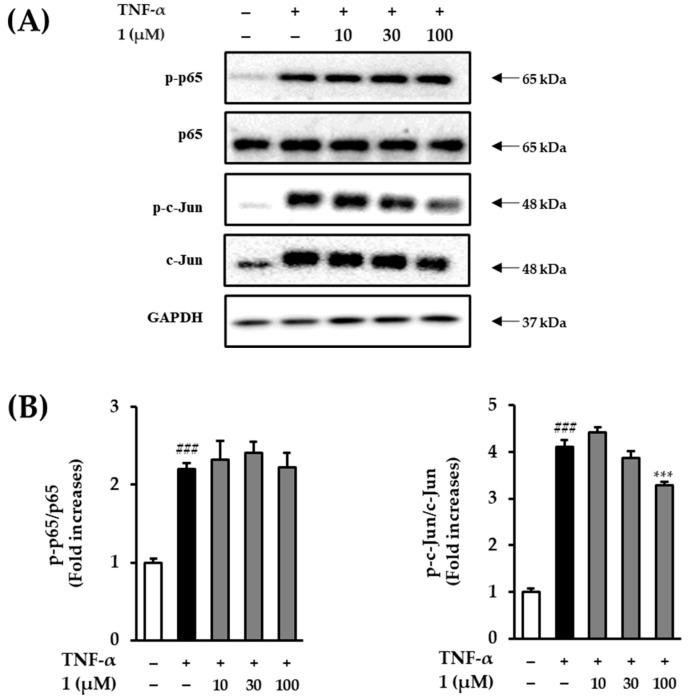
Effects of potentilloside A (**1**) on TNF-α-induced phosphorylation of NF-κB and c-Jun in NHDF. (**A**) Phosphorylation of NF-κB and c-Jun in NHDFs after TNF-α stimulation. The NHDFs were treated with 1 (3, 10, and 100 µM) for 1 h. The protein bands were analyzed by immunoblotting for p-p65, p65, p-c-Jun, c-Jun, and GAPDH. (**B**) The levels of p-p65 and p-c-Jun were expressed as the ratio of phosphorylated proteins to the corresponding total proteins. Data are presented as the mean ± SD (*n* = 3). Densitometric analysis. ^###^ *p* < 0.001versus non-treated group. *** *p <* 0.001 versus TNF-α-exposed group.

To explore the possible protective mechanism of potentilloside A (**1**) in TNF-α-treated NHDF, we examined the expression levels of proteins in TNFα-stimulated intracellular pathways, NF-κB and c-Jun. As shown in Figure 9, increased NF-κB phosphorylation was observed in the NHDFs exposed to TNF-α. Compound **1** did not decrease NF-κB phosphorylation compared with that of the TNF-α-treated group. TNF-α treatment increased c-Jun phosphorylation by 4.12 ± 0.13-fold (*p* < 0.001), and treatment with 100 μM potentilloside A (**1**) significantly decreased it by 3.28 ± 0.08-fold (*p* < 0.001).

## 4. Discussion

The skin is the outermost and largest organ that amounts to 12–16% of total body weight. The skin protects internal organs against harmful external factors as a barrier. The skin has three functional layers: epidermal, dermal, and subcutaneous [14,15]. The dermis is the main layer that supplies nutrients and moisture to the epidermis [16]. The dermis consists of the ECM, which is mainly composed of collagen, elastin, and fibronectin [17]. Among the collagen types, type 1 collagen is the most important and abundant, accounting for 80–90% of the total collagen content in the ECM [18]. Type 1 collagen is destroyed by aging-related proteins, such as MMPs. MMPs destroy the skin layer by dividing gelatinase, collagenase, and stromelysin [19]. MMP-1 is collagenase-1 that destroys collagen, such as collagen types 1 and 3 [20]. Therefore, the inhibition of MMP-1 and increased type 1 collagen prevents skin aging.

Skin aging is divided into intrinsic and extrinsic aging. Intrinsic aging are caused by endogenous factors, such as gene mutations, cellular metabolism, and hormone environment. These aging factors makes the skin to be thin, smooth, dry, and unblemished, with some loss of elasticity [21]. Extrinsic aging is promoted by exogenous factors, such as chemicals, toxins, pollutants, UV, and ionizing radiation [22]. These factors damage the skin and accelerate aging. Continuous damage to the skin causes visible symptoms (pigmentation, ptosis, and wrinkles) and cutaneous diseases (skin cancer and melanoma) [23,24]. UV radiation causes cutaneous tissue damage that promotes cytokine release, such as TNF-α and interleukins, via the activation of TNF-α receptors on the cutaneous cell surface [25]. TNF-α is an inflammatory cytokine that is a major mediator of inflammatory reactions in the skin. TNF-α upregulates MMP-1 and downregulates collagen in dermal fibroblasts [26]. TNF-α triggers ROS production and the release of proinflammatory cytokines [27]. TRAF4, a component of TNF signaling, binds to the NADPH oxidase complex. This leads to ROS generation and triggers downstream pathways [28]. ROS play an important role in aging diseases, including psoriasis, rheumatoid arthritis, and Parkinson’s disease [29]. ROS activate MMP-1 secretion through age-related signaling pathways, such as MAPKs, NF-κB, and AP-1 [30]. Thus, the inhibition of TNF-α and ROS is the key to preventing skin aging.

Previously, we found that flavonol or flavone glucuronides derived from *P*. *chinensis* effectively inhibited MMP-1 generation. Therefore, we examined the structure–activity relationship of these flavonoid glucuronides at various concentrations (Figure 4). The involvement of the functional group at position 3′ was observed at 100 μM MMP-1 secretion inhibitory activity. When comparing **3**, which is quercetin-3,7-di-glucuronide, with **5**, which is kaempferol instead of quercetin, it was observed that **5**, which lacks a hydroxyl group at position 3′, exhibited a stronger effect. This trend was similarly observed for flavonone-7-*O*-glucuronide structures, specifically between **6** and **7**.

**Figure 10:**
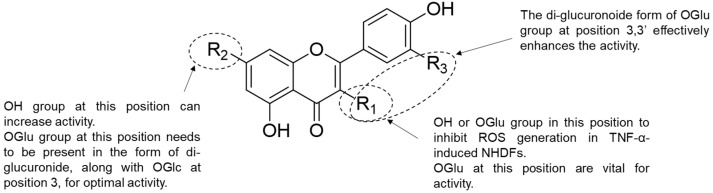
Plausible structure–activity relationship of flavonoid glucuronides in their inhibitory effect on MMP-1 secretion (at 100 µM) in TNF-α-induced NHDF.

In addition, when the backbone consisted of isorhamnetin (methoxy at position 3′) and quercetin (hydroxy at position 3′), quercetin was found to be more active than isorhamnetin in the 3,7-di-glucuronide (**3** and **4**) and 3-*O*-glucuronide structures (**8** and **9**). Furthermore, through a comparison of the di-glucuronide and mono-glucuronide structures, it was demonstrated that the inhibitory effect increased when the glucuronide was attached to the C-3 only, rather than when it was attached to both the C-3 and C-7. As a result, it was confirmed that the inhibitory effect increased when the functional group was not attached to position 3′ (Figure 10). It was also confirmed that the lack of a functional group at position 3′ and the attachment of only one glucuronide (attached C-3) increased the MMP-1 secretion inhibitory effect.

However, there was an exception to this trend for quercetin-3,3′-*O*-di-glucuronide, known as potentilloside A (**1**), which exhibited a higher inhibitory effect on MMP-1 secretion than quercetin-3-*O*-glucuronide (**8**).

In the evaluation of increased COLIA1 secretion, compounds **1**, **5**, and **11** exhibited significant effects at concentrations of 1, 3, 10, and 30 μM, with compound **1** showing the highest efficacy among them. Additionally, compounds **5**, **6**, and **9** induced a notable increase in COLIA1 secretion at a concentration of 100 μM. Although no specific structural requirements were observed for the increased secretion of COLIA1, compound **1** was confirmed to be an effective agent. Therefore, potentilloside A (**1**) exhibited the most effective reduction in MMP-1 secretion and simultaneous increase in COLIA1 secretion. However, further investigations with a larger number of samples are needed to provide evidence of the structure–activity relationship.

Potentilloside A was found to be the most effective isolate among the others. Previous studies were conducted to identify the signaling pathways involved. Similar to our results, flavonoid compounds were also shown to suppress ROS generation and MMP-1 expression. Additionally, flavonoid compounds have been shown to increase COLIA1 expression in human skin cells. Hesperidin and hesperetin were shown to inhibit MMP-1 production in physiologically aged NHDFs [31]. In addition, tectorigenin was shown to suppress ROS generation and MMP-1 secretion in UVB-damaged HaCaT cells. In addition, tectorigenin was shown to increase type 1 collagen expression in UVB-damaged HaCaT cells [32]. Quercetin 3-*O*-α-l-rhamnopyranosyl-(1″′ → 6″)-β-d-galactopyranoside, hyperin, afzelin, and cryptochlorogenic acid methyl ester were shown to inhibit MMP-1 expression in UVB-irradiated human dermal fibroblasts (WS-1 cells). These three compounds were found to increase the secretion of pro-collagen type 1. Among these, hyperin was shown to reduce ROS generation [33]. These previous studies support the hypothesis that *P. chinensis* isolates can prevent aging by inhibiting MMP-1, ROS generation, and collagen degradation.

TNF-α-induced ROS generation downregulates several signaling pathways, including MAPKs and NF-κB [34]. MAPKs regulate the transcriptional factor AP-1 complex, which is a heterodimer of c-Fos and c-Jun proteins. The activation of c-Fos and c-Jun by MAPK phosphorylation upregulates the ECM-degrading enzymes, such as MMP-1, MMP-3, and MMP-9 [20]. Figure 8 demonstrates that **1** inhibited ERK and JNK phosphorylation in TNF-α-treated NHDFs. ROS upregulate NF-κB phosphorylation in human dermal fibroblasts and keratinocytes [35,36]. In the cytoplasm, NF-κB is a heterodimeric complex composed of p65 and p50. In the nucleus, p65 phosphorylation regulates the expression of proinflammatory cytokine genes, such as COX-2, iNOS, and interleukins [37]. Figure 9 shows that potentilloside A (**1)** did not inhibit NF-κB phosphorylation in the TNF-α-treated NHDFs. These results demonstrate that potentilloside A (**1**) inhibits MMP-1 expression and secretion by inhibiting c-Jun phosphorylation via decreasing ERK and JNK phosphorylation.

Similar to our results, flavonoids were shown to inhibit ROS generation, MMP-1, and increase COLIA1 via MAPK/AP-1 or NF-κB in human skin cells. Quercetin-3-glucuronide (**8**) was reported to have skin-protective properties through the NF-κB and AP-1 pathways in human keratinocytes and melanoma cells [38].

Alpinumisoflavone inhibits ROS generation and MMP-1 secretion and expression via MAPK/AP-1 and NF-κB in TNF-α-induced NHDFs [39]. Epigallocatechin gallate (EGCG) inhibited ROS generation and MMP-1 expression via the MAPK/AP-1 pathway in UVB-irradiated NHDFs. In addition, EGCG increases COLIA1 expression via MAPK/AP-1. Eriodictyol inhibits ROS generation and MMP-1 expression in UVB-treated HaCaT cells. In addition, eriodictyol upregulates the expression of collagen type 1 genes [40].

Consequently, potentilloside A has a potential protective activity against NHDF cell damage by decreasing MMP-1 and ROS generation and increasing COLIA1 by inhibiting the phosphorylation of MAPK/AP-1. In particular, potentilloside A (**1**) exhibited stronger inhibitory effects on MMP-1 secretion and increased collagen secretion in comparison to quercetin-3-glucuronide (**8**). Additional in vivo studies are required to fully understand the activity of *P. chinensis* leaf extracts and potentilloside A as an active ingredient. Nevertheless, our study demonstrates that potentilloside A is a representative active ingredient in *P. chinensis* leaves that inhibits skin damage, including aging. Furthermore, potentilloside A not only holds potential as a candidate for addressing skin aging but also shows potential applications in other inflammatory skin diseases such as atopic dermatitis.

## 5. Conclusions

The protective effects of flavonol and flavone glucuronides from *P. chinensis* leaves against TNF-α-stimulated NHDF were investigated and compared with the results of three markers (ROS, MMP-1, and COLIA1). Comparing the three experimental results of each isolate, potentilloside A (**1**) showed the strongest skin cell protection effect among the isolates. Furthermore, potentilloside A suppressed the phosphorylation of MAPKs (ERK and JNK) and c-Jun. Thus, potentilloside A suppressed NHDF damage by regulating oxidative stress and collagenase MMP-1 activity under TNF-α-stimulated conditions. Although further investigations are required to understand the impact of potentilloside A, it nevertheless represents a candidate compound with the potential to mitigate skin damage.

## Data Availability

Data is contained within the article.
